# Authors' reply to the Letter to the Editor: Tissue factor and its procoagulant activity on cancer‐associated thromboembolism in pancreatic cancer

**DOI:** 10.1111/cas.15324

**Published:** 2022-03-24

**Authors:** Shiro Koizume, Satoshi Kobayashi, Wolfram Ruf, Yohei Miyagi

**Affiliations:** ^1^ Molecular Pathology and Genetics Division Kanagawa Cancer Center Research Institute Kanagawa Japan; ^2^ Pathology Division Kanagawa Cancer Center Kanagawa Japan; ^3^ Department of Gastroenterology, Hepatobiliary and Pancreatic Medical Oncology Division Kanagawa Cancer Center Kanagawa Japan; ^4^ Center for Thrombosis and Hemostasis Johannes Gutenberg University Medical Center Mainz Germany

**Keywords:** ELISA, extracellular vesicle, risk factor, thromboembolism, tissue factor

## Abstract

Tissue factor‐procoagulant activity (TF‐PCA) on cells is modified by multiple molecular mechanisms of encryption and decryption. The risk of thrombosis is higher for patients with a high tissue factor antigen level at registration as this enables patient’s blood more PCA‐high status before the onset of cancer‐associated thromboembolism (CAT). ELISA, including the Quantikine assay with validation as performed in our study, can contribute to more precise prediction of CAT.

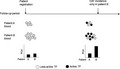

## DISCLOSURE

The authors have no conflict of interest.

Dear Editor,

We would like to address the concerns regarding tissue factor (TF) antigen assays recently raised by Mackman et al.^1^ following publication of our study.^2^

The authors conclude based on their prior work that the commercial ELISA system, Quantikine^®^ ELISA, which we used in our study, fails to measure plasma TF levels. The conclusion by Mackman et al.^1^ is based on several observations obtained with this commercial assay, which is calibrated with a soluble form of TF. First, they showed that the ELISA does not detect alternatively spliced TF, indicating that the ELISA requires a very specific sequence only present in the carboxyterminal and membrane proximal region of full‐length TF for a positive signal.^3^ They also showed that one particular diagnostic thromboplastin reagent was not recognized by the ELISA, suggesting that the TF protein conformation, which is known to be influenced by the lipid environment,^4^ may influence antigen recognition in the ELISA.

Their study also found no measurable TF antigen in plasma samples from ovarian cancer patients, whereas TF‐procoagulant activity (TF‐PCA) on isolated extracellular vesicles (EVs) from these plasmas was detectable.^3^ We studied TF antigen levels in pancreatic cancer patients, which may release forms of TF that are antigenically distinct from EV‐associated TF in ovarian cancer patients. Notably, we have previously shown that ovarian clear cell carcinoma cells express coagulation factor VII (FVII) and release TF‐FVII complexes with full activity on EV,^5^ whereas this is not the case for all cancer types. It is known that TF antibody epitopes can be hidden when FVII is associated with TF.^6^ These authors also showed that TF‐PCA increased in plasma prepared from whole blood of healthy humans after lipopolysaccharide (LPS) stimulation, but again TF antigen could not be detected by the ELISA.^3^ With this experimental set‐up, we showed that, similar to ovarian clear cell carcinoma cells, TF is released in a tight complex with FVII from the stimulated monocytes.^7^ Additionally, it is unclear whether TF‐EVs released in serous carcinoma patients are derived from cancer cells and/or immune cells.^3^ While these data indicated that the Quantikine^®^ may not detect all forms of TF‐EVs, it does not invalidate our measurements of pancreatic cancer‐derived TF‐EVs.

**FIGURE 1:**
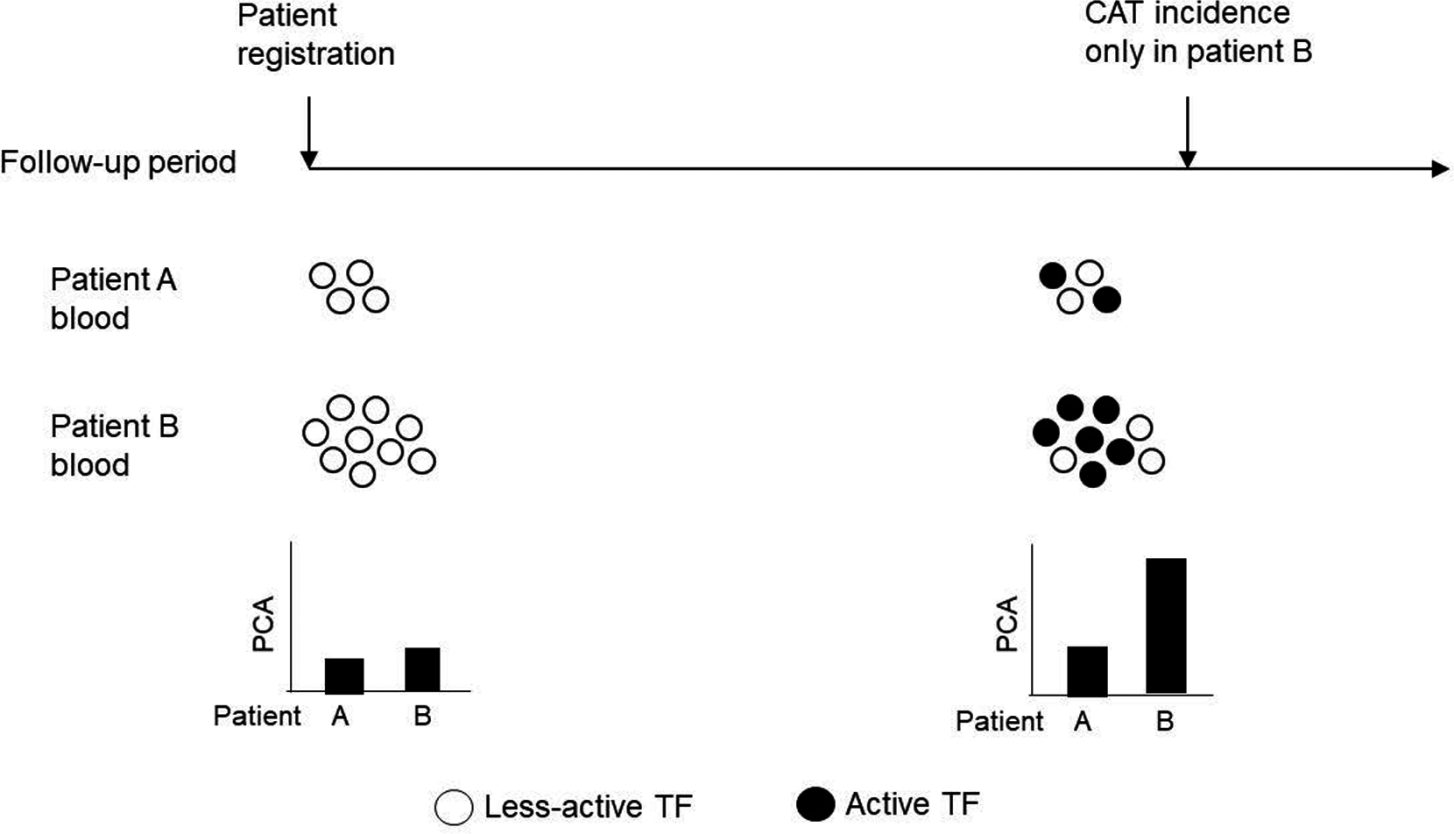
A hypothesis of cancer‐associated thromboembolism (CAT) incidence in pancreatic cancer patients with higher plasma tissue factor (TF) antigen. This illustrates how TF and its activity in the blood contributes to CAT incidence during the follow‐up period. PCA, procoagulant activity

We carefully selected the ELISA kit in our study,^2^ as we observed that TF levels in various cancer cells evaluated by western blotting are dependent on the anti‐TF antibodies used. We chose the Quantikine^®^ kit because of the strong proportional relationship between TF antigen levels in nondenatured cell lysate evaluated by this ELISA and those evaluated by western blotting using the TF 10H10 antibody and also TF mRNA levels evaluated by RT‐PCR (figures 1, S1, and S3 in Ref. [2]). By using the selected Quantikine^®^ ELISA kit, we evaluated TF antigen levels in plasma of healthy volunteers and pancreatic cancer patients. We found that higher TF antigen level significantly correlated with lower fibrinogen level, shorter prothrombin time, and higher D‐dimer level,^2^ implying that the evaluated TF antigen levels reflected the coagulation status of the sample donors. These results are consistent with the general concept that pancreatic cancer patients with high TF‐EV expression are cancer‐associated thromboembolism (CAT)‐prone owing to acceleration of fibrin deposition.^8^

Mackman's research group recommends TF‐PCA assay in evaluation of TF level in blood samples because it sensitively detects TF‐EVs increased in response to cell stimulation with LPS.^1^, ^9^ We observed that TF levels evaluated by the Quantikine do not necessarily correlate with TF‐PCA in samples of healthy volunteers, and pancreatic and biliary tract (unpublished) cancer patients without CAT.^2^ This is consistent with data by Claussen et al. showing that correlation between TF‐PCA and TF antigen level in ovarian cancer samples is low.^3^ However, TF antigen levels and TF‐PCA are highly correlated in CAT + pancreatic and biliary tract (unpublished) cancer patient samples.^2^ There are many possibly mechanisms why TF‐EV antigen levels are not correlated with TF‐PCA under noninflammatory conditions.

TF‐PCA on cells is modified by multiple molecular mechanisms of encryption and decryption.^10^ This implies that TF‐PCA in blood can be changed without affecting TF levels during the follow‐up period of cancer patients for CAT. Thus, we suggest that measurement of TF‐PCA does not necessarily predict CAT. Indeed, in our prospective study^2^ we found that TF antigen level but not TF‐PCA in plasma samples of the patients at registration is a predictive factor of CAT.^2^ However, monitoring TF‐PCA may also be useful given that it can increase just before the onset of CAT.^2^ Figure 1 illustrates a hypothesis based on our study.^2^ It demonstrates that risk of thrombosis is higher for patients with high TF antigen level at registration as this enables patient's blood more PCA‐high status before the onset of CAT.

In summary, accuracy of ELISA, including Quantikine, in detection of plasma TF may vary between cancer types. However, we believe that ELISA including the Quantikine assay with validation as performed in our study can contribute to more precise prediction of CAT.
